# Pyridazinone Derivatives Limit Osteosarcoma-Cells Growth In Vitro and In Vivo

**DOI:** 10.3390/cancers13235992

**Published:** 2021-11-28

**Authors:** Aurélie Moniot, Julien Braux, Camille Bour, Christine Guillaume, Fabien Lamret, Ingrid Allart-Simon, Sandra Audonnet, Sarah Renault, Francoise Rédini, Marie Laronze-Cochard, Janos Sapi, Sophie C. Gangloff, Stéphane Gérard, Frédéric Velard

**Affiliations:** 1 EA 4691 Biomatériaux & Inflammation en Site Osseux (BIOS), SFR CAP-Santé (FED 4231), Université de Reims Champagne-Ardenne, 51 Rue Cognacq Jay, 51096 Reims, France; aurelie.moniot@univ-reims.fr (A.M.); julien.braux@univ-reims.fr (J.B.); camille.bour@univ-reims.fr (C.B.); christine.guillaume@univ-reims.fr (C.G.); fabien.lamret@univ-reims.fr (F.L.); sophie.gangloff@univ-reims.fr (S.C.G.); 2 UFR d’Odontologie, Université de Reims Champagne-Ardenne, 2 Rue du Général Koenig, 51100 Reims, France; 3 UMR CNRS 7312, Institut de Chimie Moléculaire de Reims (ICMR), Université de Reims Champagne-Ardenne, 51 Rue Cognacq Jay, 51096 Reims, France; ingrid.allart-simon@univ-reims.fr (I.A.-S.); marie.cochard@univ-reims.fr (M.L.-C.); janos.sapi@univ-reims.fr (J.S.); stephane.gerard@univ-reims.fr (S.G.); 4 Plateforme URCACyt, Université de Reims Champagne-Ardenne, 51 Rue Cognac Jay, 51096 Reims, France; sandra.audonnet@univ-reims.fr; 5 UMR_S 1238 Sarcomes Osseux et Remodelage des Tissus Calcifiés, Phy-OS, Faculté de Médecine, Université de Nantes, 1 Rue Gaston Veil, 44035 Nantes, France; sarah.ranault@univ-nantes.fr (S.R.); francoise.redini@univ-nantes.fr (F.R.); 6 Campus Moulin de la Housse, UFR Sciences Exactes et Naturelles, Université de Reims Champagne-Ardenne, Chemin des Rouliers, 51680 Reims, France; 7 UFR de Pharmacie, Université de Reims Champagne-Ardenne, 51 Rue Cognacq Jay, 51096 Reims, France

**Keywords:** osteosarcoma, pyridazinone, cytotoxicity, migration, tumor growth

## Abstract

**Simple Summary:**

There is a dire need for novel therapeutic interventions to treat osteosarcoma. Pyridazinone derivatives have proven some efficacy in several cancer models, but their effect on osteosarcoma is yet to be evaluated. Our goal was to synthesize and evaluate, both in vitro and in vivo, some pyridazinone derivatives to provide a proof of concept of their potential as anti-osteosarcoma molecules. We demonstrated that our newly synthesized pyridazinone scaffold-based molecules might be hit-candidates to develop new therapeutic avenues for multi-therapy purposes.

**Abstract:**

Osteosarcoma is a rare primary bone cancer that mostly affects children and young adults. Current therapeutic approaches consist of combining surgery and chemotherapy but remain unfortunately insufficient to avoid relapse and metastases. Progress in terms of patient survival has remained the same for 30 years. In this study, novel pyridazinone derivatives have been evaluated as potential anti-osteosarcoma therapeutics because of their anti-type 4 phosphodiesterase activity, which modulates the survival of several other cancer cells. By using five—four human and one murine osteosarcoma—cell lines, we demonstrated differential cytotoxic effects of four pyridazinone scaffold-based compounds (mitochondrial activity and DNA quantification). Proapoptotic (annexin V positive cells and caspase-3 activity), anti-proliferative (EdU integration) and anti-migratory effects (scratch test assay) were also observed. Owing to their cytotoxic activity in in vitro conditions and their ability to limit tumor growth in a murine orthotopic osteosarcoma model, our data suggest that these pyridazinone derivatives might be hit-candidates to develop new therapeutic strategies against osteosarcoma.

## 1. Introduction

Primary osteosarcoma is a rare bone cancer mostly occurring in children and adolescents, with a second incidence peak in older patients (over 65 years) [1]. Osteosarcoma incidence in young patients is about 0.4/100,000 per year [2]. With the introduction of chemotherapy in the 1980s, the 5 year survival rate increased by 40% [3]. Treatments for osteosarcoma often consist of combining surgical resection and chemotherapy. Nevertheless, 40% of patients still suffer from relapse. Among those relapsing, 88% exhibit metastases combined with local relapse [3,4]. Lungs represent the most common metastatic site for osteosarcoma, thus resulting in low clinical outcomes with 45% 5 year overall survival for patients exhibiting metastatic complications, which are considered the major cause of deaths in these patients [5]. Of note, there has been no significant improvement in treatment options for these patients since the 1990s [6]. Because of the low efficiency of the current treatments in osteosarcoma patients, novel therapeutic approaches are needed.

Cyclic adenosine monophosphate (cAMP) is a cellular second messenger that is tightly regulated by its synthesis by adenylyl cyclase from ATP and its hydrolysis into AMP by phosphodiesterases (PDE) [7]. The functions of cAMP are mainly to modulate cell proliferation and migration and regulate cell death [8,9,10]. Currently, 11 PDE families are known, but only PDE4, PDE7 and PDE8 are cAMP specific [11]. Pyridazinone derivatives, through their ability to target PDE4, exert many potential activities [12,13] such as anti-inflammatory, anti-viral, anti-microbial and anti-obesity as well as anti-cancer activity [14,15,16,17]. The latter is a result of their in vitro anti-proliferative activity in many types of carcinoma cells (breast cancer, non-small cell lung cancers, ovarian cancer, colon cancer, glioblastoma, melanoma and leukemia) [18,19,20,21]. However, to our knowledge, only one study has reported the effect of pyridazinones on sarcomas [22]. Using an embryonal rhabdomyosarcoma model, this study showed that zardaverine was able to induce cell growth arrest and cell death, indicating that molecules possessing a pyridazinone scaffold may be useful in the treatment of osteosarcoma. Several reports have demonstrated that PDE4 inhibitors reduce bone loss and increase bone formation and mineralization [23,24,25], driving bone turnover through a neosynthesis process that may help a patient to quickly recover after surgery. Despite such arguments, the role of PDE4 inhibitors on proliferative and migratory properties of human osteosarcoma cells has not been reported.

In our study, four human osteosarcoma cell lines (Saos-2, MG-63, K-HOS and HOS-MNNG) and one murine osteosarcoma cell line (MOS-J) were used. They exhibit proliferative and migratory capabilities that are the hallmark of malignancy [26,27]. Four molecules possessing a pyridazinone scaffold, namely compounds **3a**, **3c** [28], **4aa** and **4ba** [29], derived from the first two molecules, were chosen as therapeutic candidates for their ability to inhibit PDE4, to be used against primary osteosarcoma. Our in vitro biological assays revealed that these pyridazinone derivatives were able to limit cell proliferation, survival and/or migration properties in a cell line-dependent manner. In an in vivo murine orthotopic osteosarcoma model, we demonstrated that these drugs decrease tumor development.

## 2. Materials and Methods

### 2.1. Media and Reagents

Forskolin and IBMX were from Abcam (Paris, France). Trypsin-EDTA, Dulbecco’s Modified Eagle Medium (DMEM) low glucose + Glutamax^TM^, Roswell Park Memorial Institute Medium 1640 + Glutamax^TM^ (RPMI), Dulbecco’s Phosphate Buffer Saline (DPBS), Penicillin–Streptomycin (Pen Strep, 10,000 U/mL) and Phalloidin–alexaFluor^TM^ 488 were purchased from Life Technologies (Saint Aubin, France). Bovine Serum Albumin (BSA), Dimethylsulfoxyde (DMSO), paraformaldehyde, Cell Proliferation Reagent WST-1, Bafilomycin A1, Tris(hydroxyméthyl)aminomethane (TRIS), ethylenediaminetetraacetate (EDTA), phenylmethylsulfonyl fluoride (PMSF), HEPES, acrylamide, forskolin, porcine skin gelatin, hydrochloric acid (HCl), polyethylene glycol 300, Tween 80, Anilin Blue, hematoxylin, Fuschin and calcium chloride (CaCl_2_) were purchased from Sigma Aldrich (Saint-Quentin Fallavier, France). Fetal Bovine Serum (FBS) and glycerol were from Dutscher (Issy-les-Moulineaux, France). MasterPure^TM^ DNA Purification Kit was from Epicentre^®^ Biotechnologies (Euromedex, Souffelweyersheim, France). FITC Annexin V Apoptosis Detection Kit I was from BD Pharmingen^TM^ (Le Pont de Claix, France). Triton-X100, Dithiothreiteol (DTT), SDS and Coomassie Blue G250 were from Bio-Rad (Marnes-la-coquette, France). 5-Dodecanoylaminofluorescein Di-β-D-Galactopyranoside (C_12_FDG), Pierce^TM^ BCA Protein Assay Kit, Click-It EdU Cell Proliferation Kit for Imaging, paraffin and DAPI were from ThermoFisher Scientific (Illkirch, France). cAMP ELISA kit, Ac-DEVD-AFC (Caspase-3 substrate) and 7-amino-4-trifluoromethyl coumarin (AFC) standard were from Enzo (Villeurbanne, France). Sodium chloride, acetic acid, NP40, ethanol and methanol were from VWR (Strasbourg, France). DAKO Fluorescent Mounting Medium^®^ was from Agilent (Les Ulis, France).

### 2.2. Compounds Preparation

**Figure 1:**
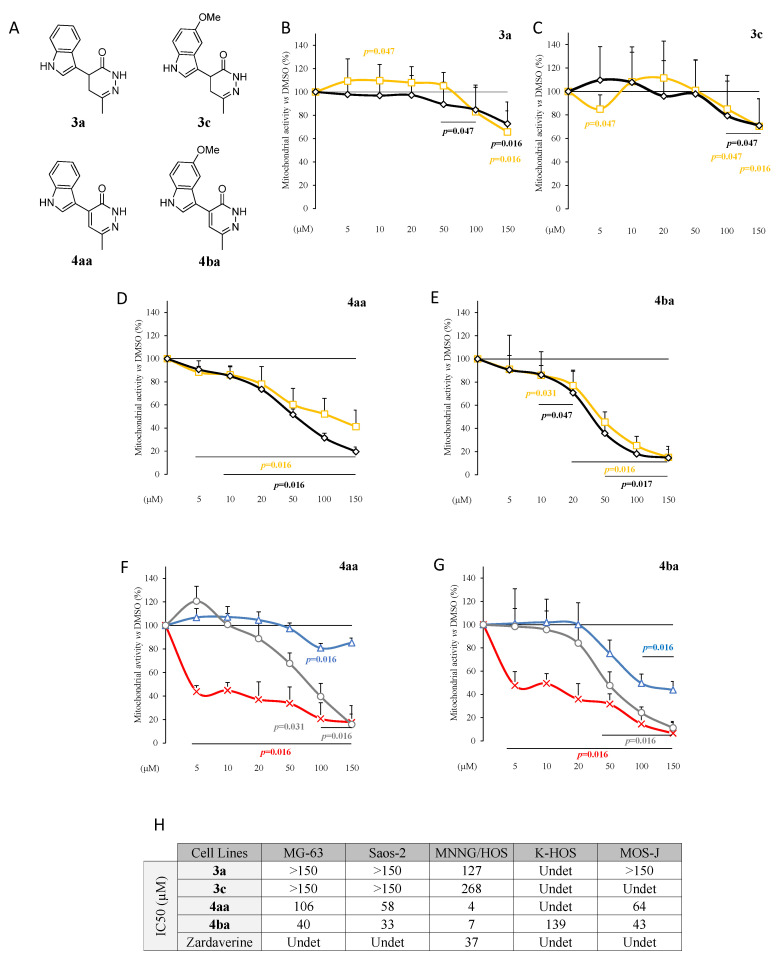
Pyridazinone derivatives exhibit cytotoxic activity. Chemical structure of compounds **3a**, **3c**, **4aa** and **4ba** (**A**). Mitochondrial activity versus DMSO on human osteosarcoma cell line Saos-2 (black diamond) and MG-63 (yellow square) after 96 h of treatment with increasing doses of **3a** (**B**) and **3c** (**C**). Mitochondrial activity versus DMSO on human osteosarcoma cell lines Saos-2 (black diamond) and MG-63 (yellow square) after 96 h of treatment with increasing doses of compounds **4aa** (**D**) and **4ba** (**E**). Mitochondrial activity versus DMSO on human osteosarcoma cell lines MNNG/HOS (red cross) and K-HOS (blue triangle) and mouse cell line MOS-J (grey circle) after 96 h of treatment with increasing doses of compounds **4aa** (**F**) and **4ba** (**G**). IC_50_ of osteosarcoma cell lines (**H**). Undetermined (Undet) means that the molecule was not efficient enough to determine the IC_50_ value. *n* = 7. *p*-values are given relative to DMSO.

Pyridazinone derivatives bearing indole or 5-methoxy-indole moieties (named **3a** and **3c**, respectively; Figure 1) were obtained by a three-step sequence starting from levulinic acid. After the preparation of the α,β-unsaturated levulinate [30], regiospecific introduction of the heterocyclic system was carried out on this intermediate by a Friedel–Crafts-type reaction, followed by condensation with hydrazine leading to the formation of the desired functionalized pyridazinones with good yields [31,32,33].

The final oxidation step using anhydrous copper chloride allowed us to obtain corresponding pyridazinone derivatives named **4aa** and **4ba** (Figure 1). For biological experiments, compounds **3a**, **3c**, **4aa** and **4ba** were solubilized in DMSO. The stock solution concentration was adapted to reach a 0.5% DMSO concentration in the culture media.

### 2.3. Cell Lines

The human cell lines Saos-2 (HTB-85^TM^), MG-63 (CRL-1427^TM^), KHOS/NP (CRL-1544™) and MNNG/HOS (CRL-1547™) were obtained from American Type Culture Collection (Manassas, VA, USA). Mouse cell line (MOS-J) was a generous gift from Dr Françoise Rédini, INSERM U1238 PhyOs, Nantes, France. Human cell lines were grown in DMEM containing 10% FBS and 1% Pen Strep. MOS-J was cultivated in RPMI with 10% FBS and 1% Pen Strep. MG-63, MOS-J, MNNG/HOS and K-HOS cell lines were finally seeded at a density of 5000 cells per cm^2^ in 96 or 24-well culture plates. Saos-2 cell line was seeded at 25,000 cells per cm^2^. All the human cells were cultured for 3 days before starting the treatments. MOS-J cell line was seeded 1 h before starting the treatments. Cell lines were used for experiments at the same passage. In all cases, cells were treated with pyridazinone-based molecules at concentrations ranging from 5–150 µM for up to 4 days because of preliminary results indicating the highest reproducibility at that time point. The medium and treatment were renewed every day. In control wells, only DMSO 0.5% was added.

### 2.4. Mitochondrial Activity

To determine optimal compound concentrations, mitochondrial activity was measured in 96-well plates using Cell Proliferation Reagent WST-1. This kit works using tetrazolium salt, which is cleaved to a soluble formazan by dehydrogenases of metabolically active cells, and the kit was used following the manufacturer’s data sheets. Tests were performed after 4 days on cells cultured independently, with medium and treatment renewal every day. Absorbance at 440 nm (WST-1) was measured on a BMG Labtech Fluostar Optima^®^ reader.

### 2.5. cAMP Measurement

After 2 h of the first treatment with pyridazinone compounds and forskolin (adenylyl-cyclase activator) at 1 µM, cells seeded in 24-well plates were lysed using 0.1 M of HCl solution with 0.1% of Triton-X100. Lysates were spun at 600 g to collect supernatant. cAMP concentration in osteosarcoma cell lines was measured by a cAMP ELISA kit following the manufacturer’s instructions. All measurements were performed at 405 nm, corrected at 580 nm on a BMG Labtech Fluostar Optima^®^. cAMP quantity was normalized to total protein content, and results were expressed compared to control.

### 2.6. Protein Quantification

Total protein content was measured using the Pierce BCA Protein Assay Kit following the manufacturer’s protocol. All measurements were performed at 562 nm on a BMG Labtech Fluostar Optima^®^.

### 2.7. Cell Count and Morphological Parameters

For the study of cell count and cellular morphology, cell lines were cultured on glass coverslips in 24-well plates for 4 days, rinsed in PBS and then fixed in 4% paraformaldehyde at 37 °C for 15 min. After fixation, cells were permeabilized using 0.1% Triton-X100 in PBS for 15 min. Staining was performed with 1/100 Phalloidin–AlexaFluor^TM^ 488 in PBS with 0.5% BSA, incubated in the dark for 30 min. After being rinsed in PBS, cells were counterstained with DAPI (1/10,000 in distilled water for 5 min). Coverslips were mounted on a glass slide using one drop of DAKO Fluorescent Mounting Medium^®^ after a last rinse in PBS. After drying overnight at room temperature, image acquisitions were performed using a Zeiss Axiovert 200 M inverted microscope and coupled AxioVision^TM^ v2.8 software. An objective with a 20-fold magnification was used. The cell number was estimated by counting cells in five randomly chosen fields per condition using the ImageJ software plugin “cell counter”.

### 2.8. DNA Quantification

The DNA content assay was performed in 24-well plates after 4 days of treatment. Total DNA was isolated from culture wells according to the protocol provided in the MasterPure^TM^ DNA Purification Kit. The concentration and purity of the DNA were analyzed by spectrophotometry (Nanodrop^®^, Thermo Scientific, Waltham, MA, USA) at 260 nm (A260 = 1 is equivalent to DNA = 50 ng·µL^−1^) and using the A260/A280 ratio for protein contamination. Purity was considered acceptable with a ratio between 1.8–2.2.

### 2.9. Cell Proliferation

Proliferation was quantified using EdU integration in cells for 24 h with treatment, in 24-well plates after 3 days of treatment. Cells were labeled using Click-iT™ EdU Cell Proliferation Kit for Imaging following the manufacturer’s protocol. Coverslips were mounted on a glass slide using one drop of DAKO Fluorescent Mounting Medium^®^. After drying overnight at room temperature, observations and acquisitions were performed using a Zeiss Axiovert 200 M inverted microscope and coupled AxioVision^TM^ v2.8 software. An objective with a 20-fold magnification was used. Cell proliferation was estimated by counting cells with or without integrated EdU using the ImageJ software plugin “cell counter” in five randomly chosen fields per condition; at least five cells per field were analyzed.

### 2.10. Apoptosis

#### 2.10.1. AnnexinV/Pi Staining

Cell apoptosis was first assessed with AnnexinV (AV)-FITC/PI staining to detect apoptosis by flow cytometry in 24-well plates after 4 days of treatment. Cells were detached using PBS/EDTA 1 mM for 5 min at 37 °C in a 5% CO_2_ humidified atmosphere. Cells were then stained according to the manufacturer’s protocol. Cells were immediately used for flow cytometry acquisition with a BD LSRFortessa™ system (BD Biosciences, San Jose, CA, USA). AV-FITC was excited with the 488 nm excitation laser, and PI was excited with the 561 nm excitation laser. The following emission filters were used to detect the appropriate fluorescence signals: AV-FITC, 530/30 nm, and PI, 610/20 nm. At the first stage, cells were selected by a FSC-A/SSC-A gate excluding subcellular debris. On this population, single cells were selected by a SSC-H/SSC-W gate. Apoptosis cells were then identified by an AV/PI gate. Discrimination between these regions was made using unstained and mono-labeled stained controls. Ten thousand cells were collected per sample. Data analysis was performed with FlowJo software (Tree Star, Stanford University, Stanford, CA, USA). Cells in region AV−PI− represent living cells, cells in region AV+IP−, early apoptotic cells, cells in region AV+IP+ late apoptotic cells, and cells in region AV−IP+ represent those with a damaged membrane only.

#### 2.10.2. Caspase-3 Activity

Apoptosis was also quantified by measuring cleaved caspase-3 activity after 4 days of treatment in 24-well plates. The latter was determined by the cleavage of synthetic fluorogenic substrates containing the amino acid sequence recognized by caspase-3. The substrate sequence was DEVD (Asp-Glu-Val-Asp) combined with a fluorophore (7-amino-4-trifluoromethyl coumarin, AFC). Upon the cleavage of the substrate by caspase-3, free AFC fluorescence emission (505 nm) was measured after excitation at 400 nm. After treatments, cells were incubated on ice for 10 min with 200 µL of lysis buffer (Tris 10 mM, NaCl 200 mM, EDTA 5 mM, Glycerol 10%, NP40 1%, pH 7.4), then stored at −20 °C. Cell lysates were thawed, centrifuged at 10,000× *g* for 10 min at 4 °C, and supernatants were collected. Fifty microliters of supernatant was incubated for 30 min at 4 °C with 50 µL of reaction buffer (DTT 10 mM, PMSF 0.1 mM, HEPES 10 mM, pH 7.4). Fluorogenic substrate for caspase-3 (Ac-DEVD-AFC) was added at 50 µM and incubated for 2 h at 37 °C. All measurements were performed on a BMG Labtech Fluostar Optima^®^ using an excitation filter at 400 nm and emission filter at 505 nm. Fluorescence (arbitrary units) was normalized to total protein content, and results were expressed as percentage compared to control.

### 2.11. Senescence

Senescence was quantified by measuring β-galactosidase activity using flow cytometry as previously described by Debacq-Chainiaux et al. [34]. Cells were cultivated in 24-well plates for 4 days before to be alkalinized with bafilomycin A1. C12FDG was added 1.5 h before detaching cells using PBS/EDTA 1 mM during 5 min at 37 °C in a 5% CO_2_ humidified atmosphere. All the cells were resuspended in 200 µL of cold-PBS before processing. Cells were immediately used for flow cytometry acquisition with a BD LSRFortessa™ system (BD Biosciences, San Jose, CA, USA). C12FDG was excited with the 488 nm excitation laser. The following emission filters were used to detect the appropriate fluorescence signals: C12FDG, 530/30 nm. At the first stage, cells were selected by a FSC-A/SSC-A gate excluding dead cells and subcellular debris. On this population, single cells were selected by an SSC-H/SSC-W gate. Ten thousand cells were collected per sample. Data analysis was performed with FlowJo software (Tree Star, Stanford University, Stanford, CA, USA). The events within this region were depicted in a C12-fluorescein fluorescence histogram. On this histogram, the relative β-galactosidase activity was estimated using the MFI of the population. Median fluorescence intensity (MFI) was compared to non-treated cells.

### 2.12. MMPs Activity

Gelatin zymography was used to detect zymogen and active forms of MMP-9 (92–82 kDa) and MMP-2 (72–62 kDa) in cell supernatants. After 10% separation gel of acrylamide containing 0.1% of porcine gelatin in TRIS buffer (pH 8.8, 1.5 M) polymerization, 4% acrylamide compressive gel in TRIS buffer (pH 6.8, 0.5 M) was added. Proteins in media were quantified using spectrophotometry (Nanodrop^®^, Thermo Scientific, Waltham, MA, USA) at 280 nm. Twenty-five micrograms of total protein were deposited per lane. The migration was carried out in a denaturing condition (via the presence of 0.1% SDS in the gel) for 30 min at 90 V in the compressive gel and then at 180 V in the separation gel. After migration, gels were washed in 12.5% Triton X-100, 2 times for 30 min, then incubated for 18 h at 37 °C in an incubation buffer with a pH of 7.6: Tris 0.6%, NaCl 11.7% and CaCl_2_ 0.74%. Gels were then stained for 30 min with Coomassie blue G-250, 10% acetic acid and 25% methanol in water and washed with acetic acid at 10% and methanol at 20% in water (2 × 30 min). The digitalization of the gels was performed with a Vilber Lourmat imaging system CN-UV/WL system controlled by the BioCapt^®^ software. The gelatin degradation bands after subtracting background were semi-quantified using the ImageJ Gel Analyzer module.

### 2.13. Cell Migration

Cell lines were seeded in 24-well plates, and scratch assays were performed as previously described [35]. At confluence, a scratch with a sterile 10 µL pipet tip was made in each well. Cells were washed once with PBS and incubated with the different compounds. Image mosaics were taken from the middle of the well immediately after adding the compound and every 20 min during 24 h with an inverted digital phase-contrast microscope (Axiovert 200 M Zeiss). The mean distance of the edges of the scratch was calculated from four measures per condition with ImageJ software. The migration speed of cells was calculated as (Distance between edges at time 0 h- distance between edges at time 24 h)/24]/2 or, when total closure of the wound occurred before the 24 h time point, (Distance between edges at time 0 h/time for total closure)/2. Wound closure percentage was calculated as 100—(Scratch area at 24 h/Scratch area at 0 h × 100).

### 2.14. In Vivo Experiments

Animal experiments were performed on mice housed in the Experimental Therapeutic Unit (Medical School, Nantes, France; agreement number D-44045) in accordance with protocols approved by the Regional Ethics Committee on Animal Experimentation (CEEA PdL 6) and the Ministry of Agriculture, under the direction of investigators certified for animal experiments. Four-week-old male C57BL/6J mice from Janvier Labs (Le Genest-Saint-Isle, France) were used. For each experiment, one week of adaptation to the environment was required before starting the manipulation, with the mice being randomized into different cages.

First, drug safety was evaluated. Because of the viscosity and toxicity of pure DMSO, molecule **4ba** was injected twice a week in intraperitoneal at 10 mg/kg and 50 mg/kg in 10% DMSO, 40% PEG300, 5% TWEEN-80 and 45% of NaCl for 4 weeks. Tolerance was evaluated by measuring the weight of mice twice a week and evaluating mice behavior daily.

The effect of compound **4ba** was then evaluated in a syngeneic orthotopic model of osteosarcoma. The MOS-J cells were injected in a para-tibial site as described previously by Crenn et al. [36]. Seven mice were treated twice a week by the intraperitoneal injection of vehicle alone, 10 mg/kg or 50 mg/kg of compound **4ba**. The efficacy of this drug was evaluated with tumor volume measurement twice a week for 2 weeks.

### 2.15. Histology

For histology analysis, collected kidneys, liver and lungs were fixed in formaldehyde 4%, washed and dehydrated by gradually replacing water in the sample with alcohol, and then alcohol was replaced by xylene. Sample were then embedded in paraffin. Multiple serial sections of the samples were made at 3.5 µm intervals, and they were rehydrated and stained with aniline blue, hematoxylin and fuchsin. Sections were mounted with a coverslip, and observations and acquisitions were performed using a Zeiss Axiovert 200 M inverted microscope and coupled AxioVision^TM^ v2.8 software for kidney and liver and using a vs. 120 OLYMPUS and coupled VS120 software and Olyvia viewer for lungs.

### 2.16. µCT Analysis

MicroCT analysis was conducted following the quantitative analysis of bone by micro-computed tomography recommendations from Campbell and Sophocleous [37]. First, we examined the tibia and fibula bones of mice using micro computed tomography (μCT, Skyscan, Bruker Optics, Ettlingen, Germany) scans with the following settings: tube voltage, 60 kV; tube current, 0.166 mA; and voxel size, 17.9 × 17.9 × 17.9 μm^3^. Samples were isolated, fixed in ethanol (70% in Dulbecco’s Phosphate Buffer Saline, Gibco), washed then stored in ethanol solution at 4 °C and scanned directly in that solution. Three-dimensional (3D) images were rebuilt and analyzed using the NRecon GPU version and CTAn (Bruker) software programs, respectively. Bone mineral density values were obtained using a standard regression curve generated by converting the attenuation coefficient from scans of hydroxyapatite standards with known mineral densities (0.25 and 0.75 g.cm^−3^). After 3D reconstruction, bone volumes were segmented using a global threshold of 0.163 g/cm^3^, which was set using the mean density leading to the segment bone retrieved in scans after manual segmentation. The whole bones (tibiae and fibulae) were first studied to avoid bias due to the use of regions of interest. STL files were calculated using an adaptive rendering model creation algorithm. Mean Bone Mineral Density (BMD) was assessed on whole bone. Then, a 1.8-mm-wide region of interest centered on the first cephalic third of the tibia was analyzed to complete the detailed bone study. For the cortical section, we analyzed the cortical tissue surface (TS), and in trabeculae, we analyzed the bone volume density (BV/TV) and trabeculae number (Tb.N).

### 2.17. Graphical Representation of Data and Statistical Analysis

Unless otherwise stated, data are presented as whisker plots: black bars represent first and ninth deciles, the bottom and top of the box are the first and third quartiles, and the red band inside the box stands for the median. The significance of the results was assessed with an exact non-parametric and stratified (when appropriate) Wilcoxon Mann Whitney test (StatXact 7.0, Cytel Inc., Cambridge, MA, USA). We used non-parametric statistics owing to a lack of normal distribution of the assessed variables. Stratification allowed the impact of individual variability to be considered. Differences were considered significant at *p* < 0.05.

## 3. Results

### 3.1. Pyridazinone Scaffold-Based Compounds Decrease Human Osteosarcoma Cell Line Metabolic Activity

To fight against osteosarcoma, one of the first expected effects is to kill cancer cells or at least to stop or reduce their proliferation. Therefore, our first approach was to determine for each tested compound the efficient concentrations able to reduce the mitochondrial activity of human Saos-2 and MG-63 osteosarcoma cells. Zardaverine, which is a PDE-4 inhibitor considered as a scaffold reference, was also tested (Figure S1). Using the compound **3a** (Figure 1A), a slight, although significant, concentration-dependent decrease of Saos-2 mitochondrial activity was observed compared to DMSO condition, and at the highest concentration (150 µM) for MG-63 (Figure 1B). Compound **3c** (Figure 1A) also induced a slight significant concentration-dependent decrease of this activity in Saos-2 and in MG-63 cultures, with a biphasic effect in the latter (Figure 1C). Compounds **4aa** and **4ba** (Figure 1A) effects pointed out a significant decrease in cell mitochondrial activity compared to the DMSO condition on both cell lines (Figure 1D,E).

Since compounds **4aa** and **4ba** were 10 times more effective than derivatives **3a** or **3c**, both were assessed on two other human cell lines able to induce tumorigenesis in vivo (MNNG/HOS and K-HOS) and on one murine cell line (MOS-J). Both compounds reduced mitochondrial activity in all cell lines, with the highest potency in MNNG/HOS (MNNG) cells, followed by the MOS-J and the K-HOS cells (Figure 1F,G).

Based on these results, the half maximal inhibitory concentrations (IC_50_) were determined. On human cell lines, **4aa** and **4ba** sufficiently impacted cell mitochondrial activity to determine IC_50_ values (Figure 1H). The lowest IC_50_ values were observed on Saos-2 and MNNG with both compounds. Since Saos-2 and MNNG are the most sensitive cell lines, they were chosen for subsequent experiments, using 10 µM and 50 µM as effective concentrations (Figure 1D–G), close to the determined IC_50_. All these results indicated that pyridazinone compounds were able to decrease human osteosarcoma cell lines’ mitochondrial activity.

### 3.2. Pyridazinone Scaffold-Based Compounds Increase Intracellular cAMP

**Figure 2:**
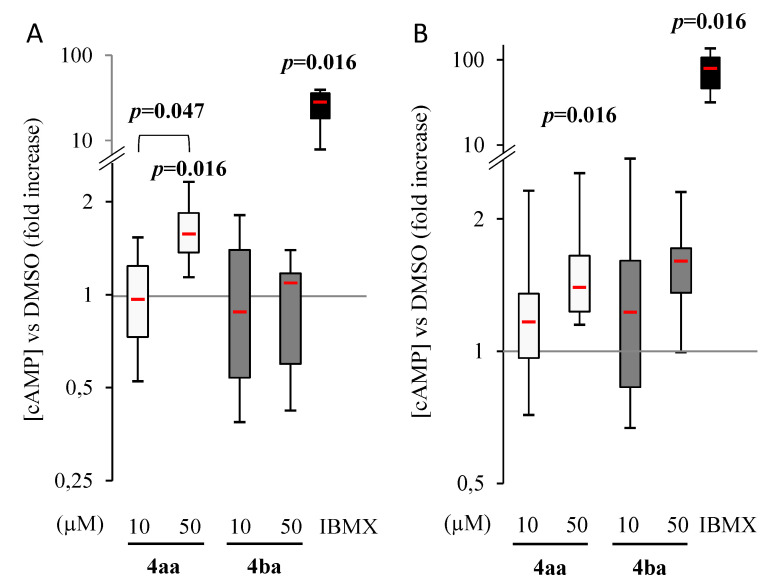
Pyridazinone scaffold-based molecule **4aa** increases intracellular cAMP. Intracellular cAMP versus DMSO on human osteosarcoma cell lines Saos-2 (**A**) and MNNG/HOS (**B**) after 2 h of treatment with compounds **4aa**, **4ba** or IBMX. *n* = 7. *p*-values are given relative to DMSO on box-plots; the brackets indicate the conditions compared.

As compounds **4aa** and **4ba** have been intended to be PDE4 inhibitors, intracellular cAMP was quantified in Saos-2 and MNNG cell lines after 2 h of treatment. Once treated with pyridazinone compounds, the intracellular cAMP concentration was compared to control. IBMX treatment, which is a non-selective PDE inhibitor, was used as a positive control. Zardaverine was also tested (Figure S2A) and found to induce cAMP increase in Saos-2 and MNNG. Compound **4aa** increased intracellular cAMP in both cell lines at 50 µM treatment, whereas changes induced by compound **4ba** were not significant (Figure 2A,B). The panel of cAMP-selective PDE expression was assessed in both cell lines and shown in Figure S2B.

### 3.3. Pyridazinone Scaffold-Based Compounds Decrease Osteosarcoma Cell Line Proliferation

**Figure 3:**
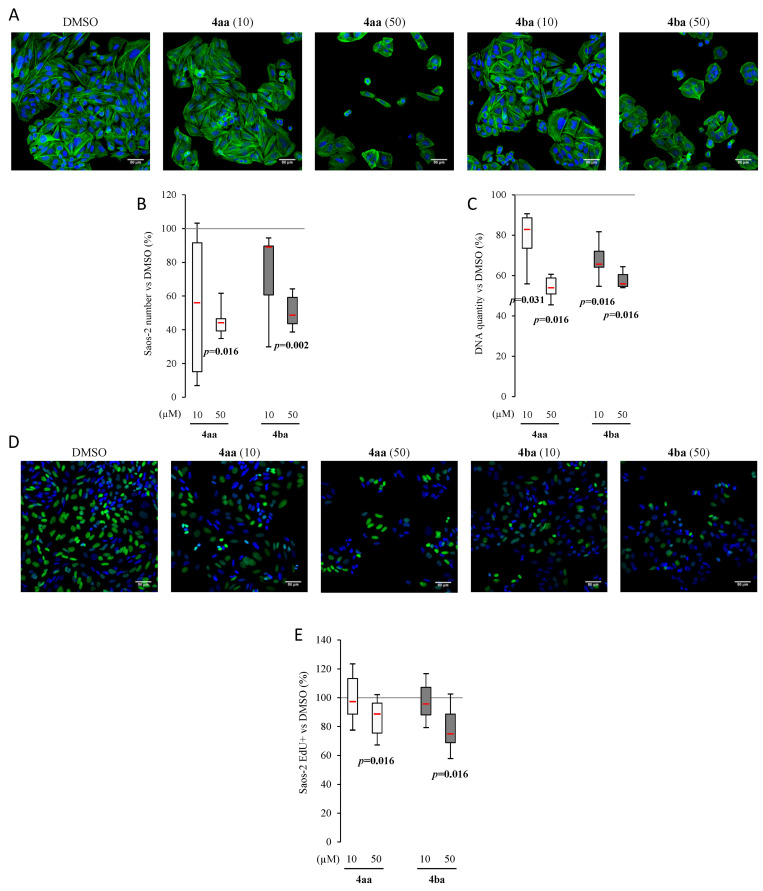
Pyridazinone scaffold-based molecules decrease Saos-2 proliferation. Representative confocal images of cell morphology using confocal LSM 710 NLO Zeiss. Cytoskeleton was stained with Phalloidin/Alexafluor^TM^488 (green) and nuclei with DAPI (blue) (**A**). Scale bar = 50 µm. Saos-2 number variation versus DMSO with cell count per field (**B**) and DNA quantification (**C**). Representative confocal images of cell proliferation with EdU stained (green) and nuclei stained with DAPI (blue) (**D**). Scale bar = 50 µm. Number of EdU positive cells quantification versus DMSO (**E**). *n* = 7, min 8 cells per field, randomly chosen, 5 fields per condition. *p*-values are given relative to DMSO on box-plots.

As a decrease of metabolic activity of human osteosarcoma cell lines after a 96 h-treatment period was observed, the change in cellular morphology was assessed using fluorescence microscopy (Figure 3A). In control condition (DMSO), Saos-2 cells were well attached and fully spread, exhibiting a tensile cytoskeleton and a characteristic polygonal shape. Compounds **4aa** and **4ba** did not display any morphological effects at the single-cell level but induced a concentration-dependent reduction in cell density. The cytotoxicity was also evaluated by cell count on microscope fields. As a result, a significant decrease was observed after incubation with compounds **4aa** and **4ba** at 50 µM (Figure 3B). To confirm the cell count results, end point DNA measurements were also performed after 96 h of treatment. In Figure 3C, the results confirmed that Saos-2 cell line was sensitive to pyridazinone scaffold-based molecules, with a decrease in cell culture DNA content after treatment with compounds **4aa** and **4ba** compared to the DMSO condition. Following EdU incorporation, compounds **4aa** and **4ba** clearly showed a significant inhibition of Saos-2 cell proliferation after the 96 h treatment period at 50 µM only (Figure 3D,E).

**Figure 4:**
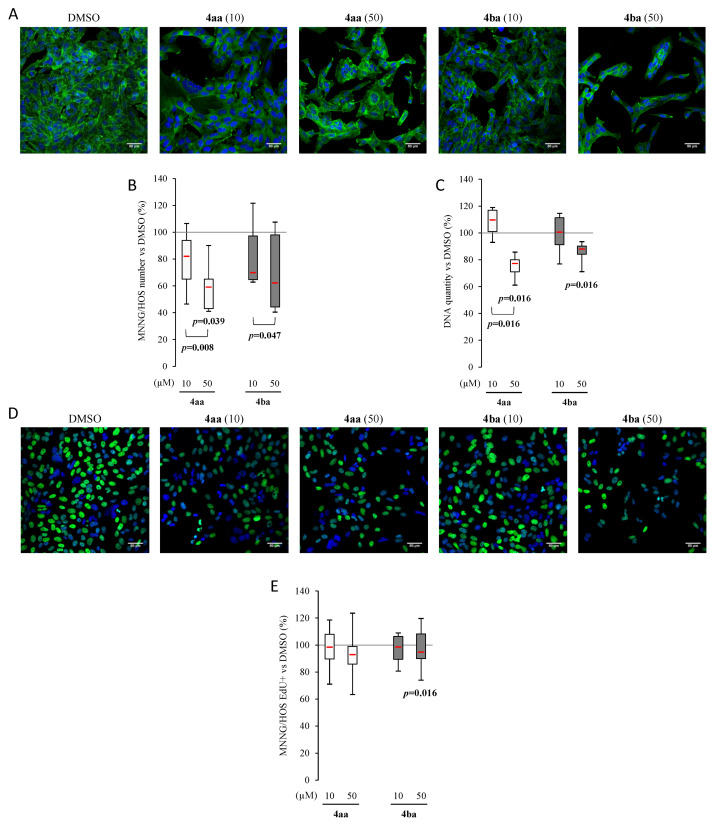
Pyridazinone scaffold-based molecules decrease MNNG/HOS proliferation. Representative confocal images of cell morphology using confocal LSM 710 NLO Zeiss. Cytoskeleton was stained with Phalloidin/Alexafluor^TM^488 (green) and nuclei with DAPI (blue) (**A**). Scale bar = 50 µm. MNNG/HOS number variation versus DMSO with cell count per field (**B**) and DNA quantification (**C**). Representative confocal images of cell proliferation with EdU stained (green) and nuclei stained with DAPI (blue) (**D**). Scale bar = 50 µm. Quantification of EdU positive cells versus DMSO (**E**). *n* = 7, min 8 cells per field, randomly chosen, 5 fields per condition. *p*-values are given relative to DMSO on box-plots; the brackets indicate the conditions compared.

In parallel, the same experiments were performed on MNNG cells. In control conditions, a very dense monolayer was observed. After treatment with compounds **4aa** and **4ba** at 50 µM, the monolayer was less dense and cells were more elongated. At this concentration, actin staining demonstrated a relocalization from a pericellular towards perinuclear location and a clear decrease of cell density (Figure 4A). This was validated by the cell count per field, showing that MNNG responded to both compounds at 50 µM (Figure 4B).

End-point DNA measurement was assessed and showed that treatment with both compounds induced a concentration-dependent decrease compared to DMSO (Figure 4C). Cell proliferation, evaluated by EdU, showed a slight reduction only with compound **4ba** at 50 µM (Figure 4D,E). Our data revealed that compounds **4aa** and **4ba** were able to decrease cell proliferation.

### 3.4. Pyridazinone Scaffold-Based Compounds Increase Osteosarcoma Cell Apoptosis

Antitumor compounds work by limiting tumor growth and decreasing tumor size by inducing cancer cell death; therefore, we tested their ability to induce apoptosis after a 96 h treatment period.

**Figure 5:**
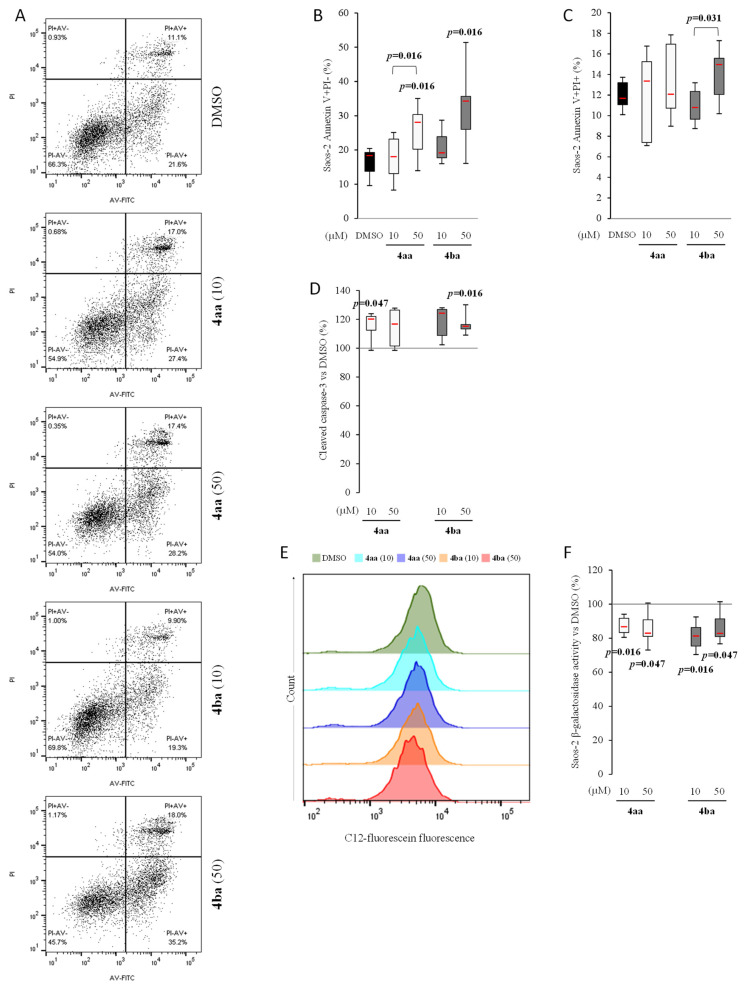
Pyridazinone scaffold-based molecules increase Saos-2 apoptosis and reduce senescence. Representative cytogram of AV and PI stain (**A**). Quantification of early (AV+/PI- cells (**B**)) and late apoptosis (AV+/PI+ cells (**C**)). Quantification of cleaved caspase-3 versus DMSO (**D**). Representative histograms of intracellular β-galactosidase activity (**E**) and its quantification versus DMSO (**F**). *n* = 7. *p*-values are given relative to DMSO on box-plots; the brackets indicate the conditions compared.

Compounds **4aa** and **4ba** induced a concentration-dependent response with a significant increase of AV+/PI- Saos-2 cells with compounds **4aa** (Figure 5A–C). These results were confirmed by cleaved caspase-3 quantification, which showed an increase at 50 µM (Figure 5D). As cell senescence induced by chemotherapy may lead to cancer therapy resistance [38], β-galactosidase (senescent cell biomarker [39]) was quantified using C12FDG. Compounds **4aa** and **4ba** significantly decreased the CD12FDG signal compared to DMSO at both concentrations used (Figure 5E,F).

**Figure 6:**
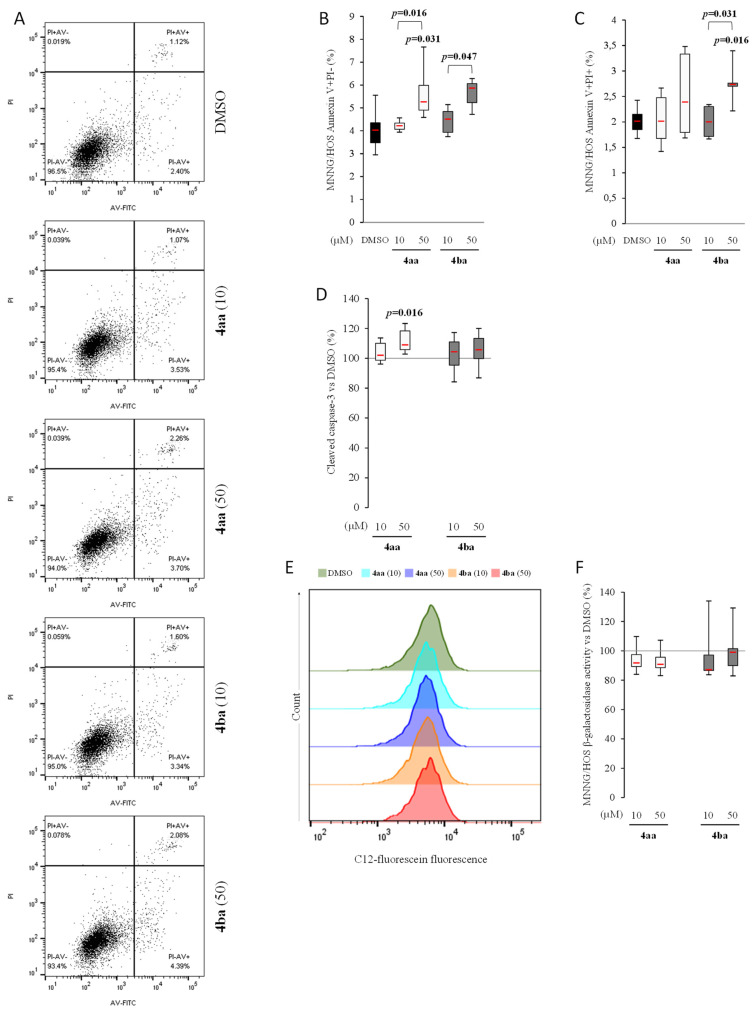
Pyridazinone scaffold-based molecules increase MNNG/HOS apoptosis and reduce senescence. Representative cytogram of AV and PI stain (**A**). Quantification of early (AV+/PI- cells (**B**)) and late apoptosis (AV+/PI+ cells (**C**)). Quantification of cleaved caspase-3 versus DMSO (**D**). Representative histograms of intracellular β-galactosidase activity (**E**) and its quantification versus DMSO (**F**). *n* = 7. *p*-values are given relative to DMSO on box-plots; the brackets indicate the conditions compared.

Comparing human osteosarcoma cell lines Saos-2 and MNNG in the DMSO condition, there were about 16% of AV+/PI- cells in the first cell line and only 4% in the second line (Figure 5A–C and Figure 6A–C). The percentage of AV+/PI- MNNG cells was increased by both compounds in a concentration-dependent manner (Figure 6A–C), inducing an increase of caspase-3 activity (Figure 6D), but with no significant effect on senescence (Figure 6E,F). These results showed that pyridazinone compounds **4aa** and **4ba** were able to increase cell death in addition to decreasing cell proliferation without inducing cell senescence.

### 3.5. Pyridazinone Scaffold-Based Compounds Reduce MNNG Migratory Capabilities In Vitro

**Figure 7:**
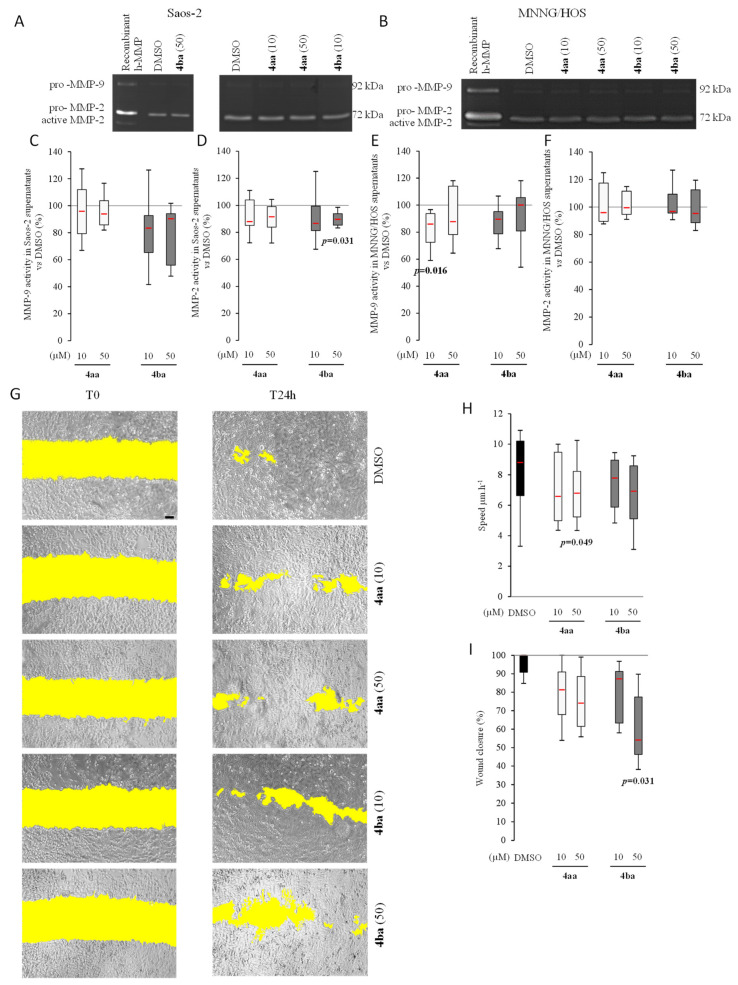
Pyridazinone scaffold-based molecules decrease osteosarcoma cell migration capability in vitro. Representative photography of gelatin zymogram of Saos-2 (**A**) and MNNG/HOS (**B**) culture supernatants. Saos-2 MMP-9 (**C**) and MMP-2 (**D**) activity quantification versus DMSOS. MNNG/HOS MMP-9 (**E**) and MMP-2 (**F**) activity quantification versus DMSO. Representative fields of MNNG/HOS migration at T0 and T24h (**G**); wound area evidenced in yellow. Scale bar = 100 µm. Quantification of cell migration speed (**H**) and wound closure percentage (**I**). *n* = 7. *p*-values are given relative to DMSO on box-plots.

The ability to promote the metastatic process is a common feature of many cancer cell lines including MNNG, but not Saos-2. This occurs mainly through extracellular matrix degradation by cells, coupled with migratory properties that ensure the dissemination of the metastatic cells. To assess the capability of compounds **4aa** and **4ba** to block metastatic potential, gelatinolytic activity was evaluated by quantifying MMP-9 and MMP-2 release using a zymography technique after the 96 h treatment period (Figure 7A,B). Saos-2 total MMP-9 secretion was not impacted by compounds **4aa** and **4ba** (Figure 7C). Regarding MMP-2 secretion, we observed a decrease with compound **4ba** at 50 µM (Figure 7D). The same profile was observed in MNNG cells, with a decrease of total MMP-9 with **4aa** (Figure 7E). Pyridazinone compounds did not impact MMP-2 release by MNNG (Figure 7F).

Then, a human osteosarcoma cell motility test was performed using scratch assays for a 24 h period. Saos-2 cells were low migrating cells as they traveled only 15 µm after 24 h. Obviously, such a weak migratory ability was not affected by the treatments (Figure S3). In contrast, MNNG cells exhibited a basal cell motility of 8 µm.h^−1^, which was significantly reduced in the presence of compound **4aa** (Figure 7G,H). Compound **4ba** also induced a trend to decrease migration with treatment at 50 µM. Wound closure percentage was also evaluated. In Saos-2, the maximal percentage of wound closure was 19% in control conditions, and the highest decrease was obtained after treatment with compound **4ba** at 50 µM (Figure S3). Regarding MNNG, a trend of decreasing with compound **4aa** was observed (Figure 7I). Treatment with compound **4ba** also decreased the wound closure at 50 µM. These results showed that pyridazinone compounds were able to decrease cell motility and migration in vitro.

### 3.6. Pyridazinone Scaffold-Based Compounds Reduce Orthotopic Tumor Growth

**Figure 8:**
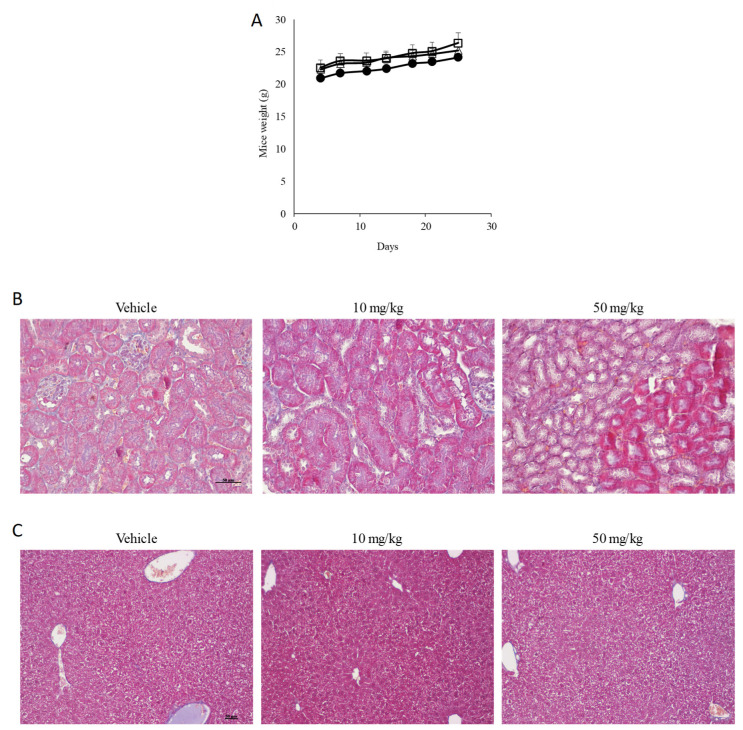
Pyridazinone scaffold-based molecules do not show toxicity in vivo in an orthotopic osteosarcoma model. Mice weight evolution in a time-dependent manner after intraperitoneal injection of vehicle (square), compound **4ba** at 10 mg/kg (triangle) and at 50 mg/kg (circle) (**A**). Representative field of histological analysis of liver (**B**) and kidney (**C**). Scale bar = 50 µm, *n* = 7.

Since our in vitro data indicated that compound **4ba** is more effective compared to compound **4aa** on the murine osteosarcoma cell line MOS-J (Figure 1H), only the effects of compound **4ba** were subsequently investigated in a murine orthotopic osteosarcoma model using MOS-J cells, as previously described [36]. First, drug safety was evaluated by the intraperitoneal injection of compound **4ba**, twice a week for 4 weeks. No differences in mouse weight were noted after the treatment with compound **4ba** compared to vehicle alone, neither at 10 mg/kg nor at 50 mg/kg conditions (Figure 8A). Furthermore, no significant change after nephritic or hepatic histological examinations was noted (Figure 8B,C).

**Figure 9:**
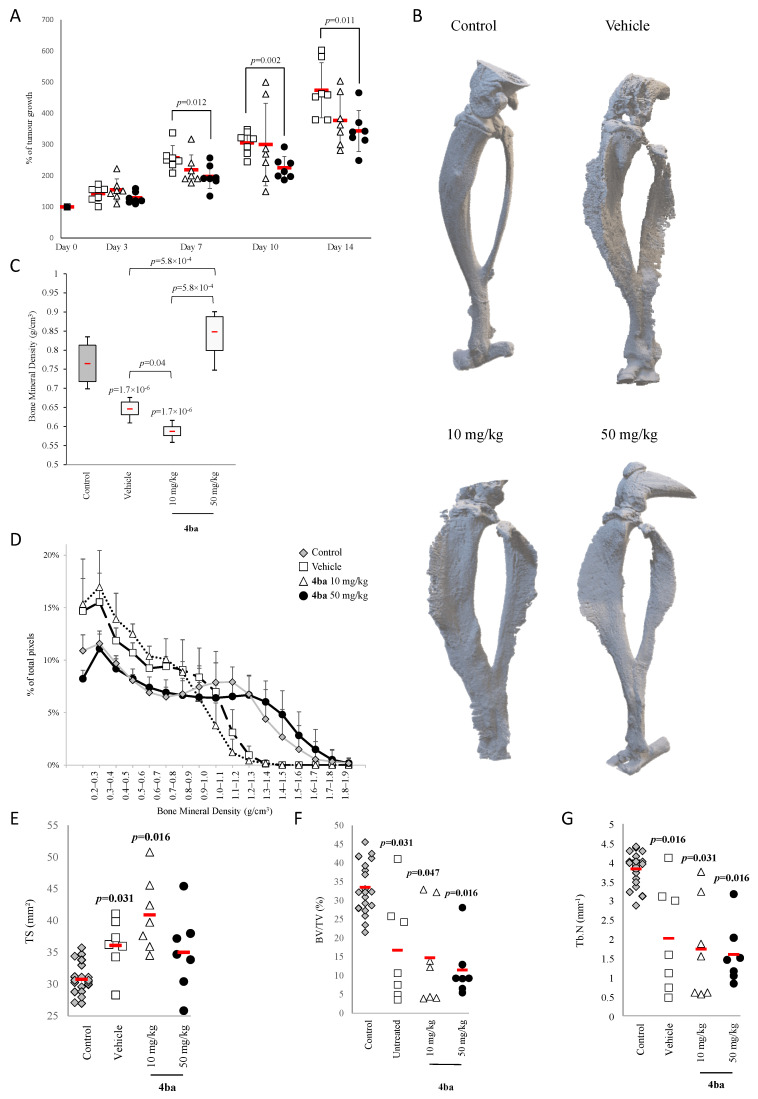
Pyridazinone scaffold-based molecules decrease tumor growth in an orthotopic osteosarcoma model. Tumor growth evolution after first day of treatment with vehicle alone (white square), with compound **4ba** at 10 mg/kg (white triangle) and at 50 mg/kg (black circle) (**A**). Representative MicroCT imaging of tumor-bearing mice for contralateral paw (control, grey diamond), paw from mice treated with vehicle (white square), compound **4ba** at 10 mg/kg (white triangle) and at 50 mg/kg (black circle) (**B**), quantification of total bone mineral density (**C**) and distribution of bone density (**D**). MicroCT quantification of local cortical surface (TS), trabecular bone volume (BV/TV) and trabecular number (Tb.N) (**E**–**G**). *n* = 21 for control and *n* = 7 for each other conditions. *p*-values are given relative to DMSO on box-plots; the brackets indicate the conditions compared.

In an orthotopic osteosarcoma model using MOS-J, compound **4ba** was administered at 50 mg/kg 2 weeks after tumor induction. We observed a significantly reduced tumor growth of 23% after 7 days of treatment and a 27% reduction compared to control after 2 weeks of treatment twice a week (Figure 9A). MicroCT analysis of tibia and fibula of mice showed ectopic bone formation around the tumor, which was decreased following a treatment with compound **4ba** at 50 mg/kg (Figure 9B). Mice treated with vehicle alone or compound **4ba** at 10 mg/kg demonstrated a decrease of bone mineral density compared to contralateral leg without tumor (control) (Figure 9C). Compound **4ba**, at 10 mg/kg, also exhibited a decrease of bone mineral density compared to vehicle alone. In contrast, compound **4ba** at 50 mg/kg induced an increase of bone mineral density compared to vehicle, restoring bone mineral density similar to the control level. Compound **4ba** at 50 mg/kg also increased bone mineral density compared to the 10 mg/kg dose (Figure 9C). Bone mineral density distribution analysis confirmed these global BMD results. Compound **4ba** at 10 mg/kg followed the same profile as the vehicle, whereas at 50 mg/kg, this molecule overlaid the profile of bone without tumor (Figure 9D).

Most relevantly, bone histomorphological parameters (cortical tissue surface (TS), trabecular bone volume (BV/TV) and trabecular number (Tb.N)) were extracted from 3D reconstructs. A significant increase of cortical tissue surface with both vehicle and compound **4ba** at 10 mg/kg was observed compared to control (Figure 9E). Values of BV/TV and Tb.N in vehicle-treated mice decreased when compaed to control conditions, and compound **4ba** did not affect these trabecular bone variables at any concentrations compared to vehicle condition (Figure 9F,G).

**Figure 10:**
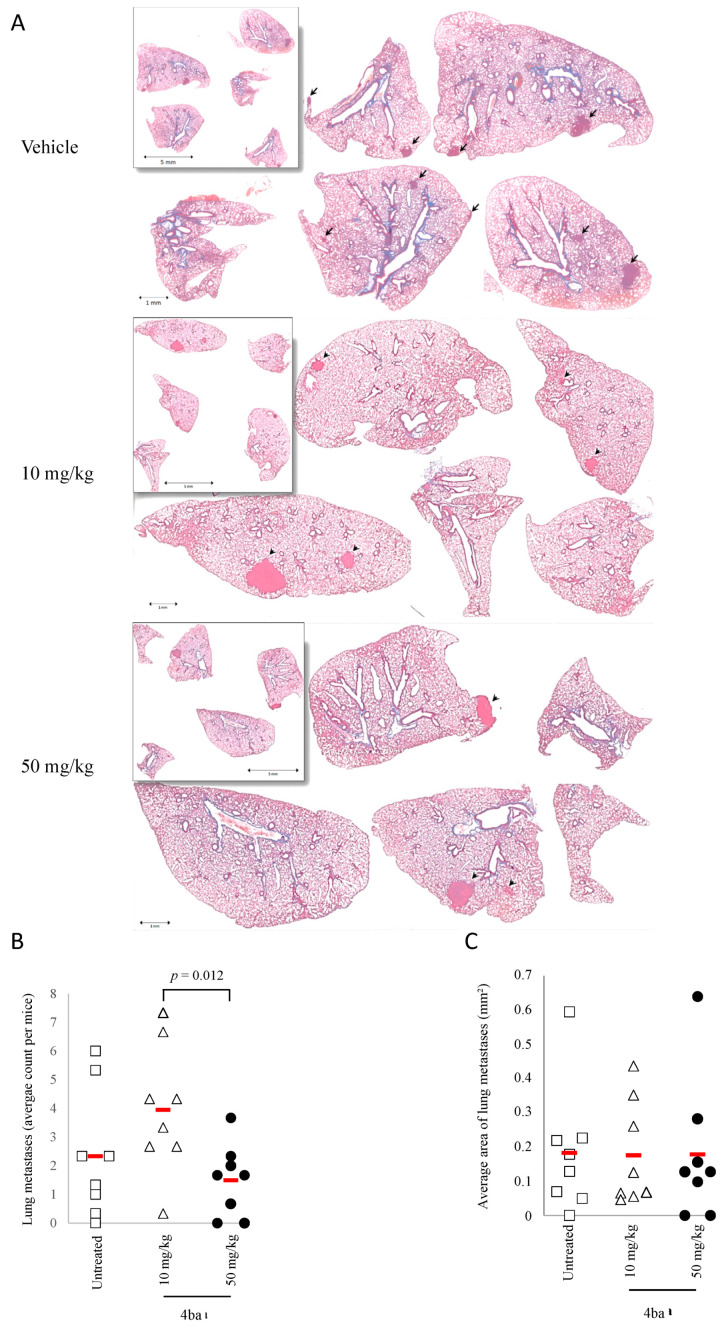
Pyridazinone scaffold-based molecule effect on lung metastases in an orthotopic osteosarcoma model. Representative field of lung metastases (**A**). Quantification of lung metastases number after treatment with vehicle (white square), compound **4ba** at 10 mg/kg (white triangle) and at 50 mg/kg (black circle) (**B**) and metastases surface (**C**). *n* = 8. The brackets indicate the conditions compared.

Compound **4ba** was efficient in limiting cell dissemination in vitro; therefore, the capability of this drug to limit lung metastases was evaluated in the same orthotopic osteosarcoma model (Figure 10A). Compound **4ba** did not decrease the lung metastasis number and area compared to vehicle at 50 mg/kg. In contrast, a paradoxical increase of metastasis number was observed at 10 mg/kg. These in vivo results showed that compound **4ba** at 50 mg/kg was able to decrease orthotopic tumor growth and to maintain bone mineral density but did not impact metastatic cell dissemination in this osteosarcoma model.

## 4. Discussion

The development of new therapeutic alternatives for osteosarcoma treatment has become pivotal to fight against relapse and improve patient survival, especially for high-risk patients with metastases or relapsed disease. In the present study, we provide evidence that pyridazinone derivatives are able to reduce osteosarcoma cell proliferation and migration in vitro, in a cell-dependent manner, and to decrease tumor growth in vivo. Preliminary data showed that **4aa** and **4ba** pyridazinone scaffold-based molecules are efficient PDE-4 inhibitors [29]. Pyridazinone compounds have already been shown to have tumor suppressor effects in many carcinomas and in one sarcoma, but to date, there are no data available for osteosarcoma [18,19,20,21,22].

Several studies have reported that anti-PDE4 molecules, leading to elevated cAMP levels, induce cell proliferation arrest and apoptosis on colon cancer cells [40]. The anti-proliferative effect of PDE4 inhibitors such as rolipram has been described on mandible osteosarcoma [41]. Regarding the literature indicating that Saos-2 cells express PDE4 [42,43] and our own data showing that MNNG cells express this enzyme, our study supports the hypothesis that anti-PDE4 molecules might be used as putative anti-cancer agents in osteosarcoma.

Different effects of compounds **3a**, **3c**, **4aa** and **4ba** were highlighted. The second generation, namely **4aa** and **4ba**, pharmacomodulated from **3a** and **3c**, respectively, was more effective than the first, allowing IC_50_ determination. Importantly, the IC_50_ varied depending on the cell line, highlighting the potential selectivity of our molecules for which the mechanisms remained to be determined.

Nevertheless, for all cell lines studied, IC_50_ values were lower for compounds **4aa** and **4ba** than for compounds **3a** and **3c**, or the anti-PDE4 reference compound zardaverine. This effect might be related to the structural modification between the two families. The additional double bond in the pyridazinone core, differentiating the two generations, could positively influence the planarity of compounds **4aa** and **4ba** (vs **3a** and **3c**), promoting interactions into the oblong hydrophobic pocket [28,29]. The different cellular response could not be fully explained by the fact that different PDE4 subunits could be expressed differently in Saos-2 and MNNG cells. Indeed, compounds **4aa** and **4ba** induced a similar increase of intracellular cAMP in both cell types, despite a differential response having been highlighted by Sun et al., who studied the anti-cancer effect of zardaverine in different hepatocarcinoma cell lines [44]. Another hypothesis relies on the fact that PDE4 inhibitors possessing a pyridazinone scaffold could exhibit anti-cancer effects independently of PDE4 activity, as demonstrated by Sun et al. Among hypotheses to investigate further, the pyridazinone-based compound inhibition of c-Met kinase, activation of ATM-Chk2 pathway and ROS accumulation and proteasome function impairment have already been suggested [45,46,47]. These could explain part of our results, since zardaverine induced a high increase of intracellular cAMP and a slight decrease in cell proliferation, contrary to the effects of compounds **4aa** and **4ba**.

Furthermore, to limit tumor cell growth, an anticancer drug needs to be able to decrease cell proliferation and/or induce cell death. The decrease of metabolic activity in a dose-dependent manner can be reflected by a decrease of cell proliferation or only a decrease of mitochondrial activity. In our study, cell morphology assessments showed a decrease of cell density after treatment with pyridazinone scaffold-based molecules. These results were confirmed by the decrease of cell number and DNA quantity in most of our conditions, suggesting reduced cell proliferation and/or cell death induction, shedding light on the activity of our new pyridazinone derivatives. In addition, the trend of a DNA increase in MNNG cells treated with **4aa** at 10 µM suggests that some other mechanisms, such as G2/M cell cycle arrest, may occur, as proposed by Gong et al. [46]. These data were further confirmed by an increase of apoptosis associated with an increase of extracellular phosphatidyl serine evidenced by annexin V binding and cleaved caspase-3 activity. Demaria et al. have reported that chemotherapy using doxorubicin may induce senescence, and many research works have shown that senescence induced chemotherapy resistance [48,49]. In our model, the β-galactosidase quantification showed equivalent senescent cells with compounds **4aa** and **4ba** and the control. Therefore, we can speculate that the cytotoxic effects of compounds **4aa** and **4ba** would not be impaired by the rise of senescence in treated cells.

Osteosarcoma is characterized by its capability to generate metastases in large numbers of patients [3,4]. Consequently, blocking cancer cell dissemination is a major concern for efficient therapy. Cell dissemination processes require matrix degradation, using metalloproteases such as MMP-9 and MMP-2. These two collagenases play a key role in basement membrane disruption and cancer cell dissemination. Oku et al. showed that MMP-9 expression and release is responsible for gastric carcinoma cell invasion [50]. In our work, compounds **4aa** and **4ba** slightly decreased metalloprotease secretion. Using Saos-2 cells, no effect of the tested anti-PDE4 compounds was observed. This could mainly be attributed to an intrinsic lack of migratory skills for the Saos-2 cells. In the present work, this has been evidenced by their lowest migratory speed (10 times less than in MNNG cells). Moreover, this observation could be a part of the explanation for the poor metastatic potential of Saos-2 previously observed [26]. However, our pyridazinone derivatives have exhibited a capability to reduce the migration of MNNG cells. Watanabe and colleagues showed that rolipram, a PDE4 inhibitor, can decrease mouse melanoma cell migration [51]. Migration depends on focal adhesion properties and cytoskeleton rearrangement, and PDE4 is necessary for actin remodeling [52]. This mechanism sheds a new light on the perinuclear relocalization of actin observed with compounds **4aa** and **4ba** at 50 µM, which could explain the efficiency of this drug in this in vitro migration model.

Regarding our IC_50_ results on osteosarcoma cell lines, compound **4ba** is more effective than compound **4aa**. Crenn et al. showed that the tumor environment influences the tissue response to chemotherapy [36]. Based on their work, we have chosen a syngeneic orthotopic model of osteosarcoma induced by the paratibial injection of MOS-J cells. This model has been shown to be particularly effective to study anti-cancer drug effects in vivo (i.e., reference molecules such as doxorubicin failed to induce significant reduction in tumor size) [36]. To model the clinical situation, the first injection of drug took place 19 days after tumor initiation by MOS-J injection. The tumor size before the first treatment ranged from 90 mm^3^ to 544 mm^3^. Despite homogeneous repartition between the groups, such a high variability may account for the lack of effect of compound **4ba** on metastases progression. Although tending to reduce the number of metastases per lung, compound **4ba** did not statistically impact metastases development. However, even in such drastic conditions, our results evidenced a decrease of tumor growth after treatment with compound **4ba** at the highest dose tested. However, a poor vascularity has been observed in this osteosarcoma model [36]. Considering the intraperitoneal administration of the treatment, one can speculate that in this condition, our pyridazinones scaffold-based molecules exhibited poor bioavailability in tumor environment. To increase the efficiency of therapeutics delivery to the tumor, bone targeting represents a promising approach, as recently described by David et al. [53]. Ectopic bone formation is a hallmark of osteosarcoma development in mice [54]. This aspect of the disease may be controlled by anti-resorptive drugs such as bisphosphonates. Nevertheless, a recent clinical trial demonstrated that zoledronate also showed detrimental effects such as hypocalcemia, hypophosphatemia or an increase of lung metastases, underlying the need for alternative molecules [55]. In the present study, we observed that compound **4ba** used at 50 mg/kg is able to limit tumor-induced ectopic bone formation, suggesting an impact on bone remodeling. In addition to reducing tumor growth and ectopic bone formation, compound **4ba** also blunted the tumor-induced decrease of bone mineral density. These data corroborate an older study evidencing the capability of PDE4 inhibitor to increase bone mineral density in a dose-dependent manner [56] and a recent review from Porwal et al. highlighting the effect of such molecules on bone formation and resorption [57]. In humans, actual methotrexate-based osteosarcoma chemotherapy shows a decrease of femoral bone density after treatment [58]. Such an adverse effect might be counteracted using therapeutics such as compound **4ba** in a multi-therapy treatment.

## 5. Conclusions

The results presented herein reinforce the interest in using pyridazinone derivatives as active molecules against cancer in bone tissue. Importantly, no study on anti-PDE4 treatment resistance and compound **4ba** limited cell senescence has been reported so far, suggesting that this molecule could be promising in some chemo-resistant cancer cells. The exact mode of action of pyridazinone compounds **4aa** and **4ba** remains to be determined. Now, it could be interesting to evaluate the efficiency of compounds **4aa** and **4ba** not only on osteosarcoma cells but also on lung and prostate-derived cancer cells, which are well known for their bone tissue tropism [59]. Our results show modest effects of compound **4ba** on osteosarcoma onset but reveal interesting features regarding bone tissue maintenance. Single-molecule chemotherapy is currently rare, and we believe that compound **4ba** might be particularly interesting for multi-therapy purposes.

## Data Availability

The data presented in this study are available on request from the corresponding author.

## Supplementary Materials

The following are available online at https://www.mdpi.com/article/10.3390/cancers13235992/s1, Figure S1: Chemical structure of zardaverine and mitochondrial activity versus DMSO on human osteosarcoma cell line Saos-2, MG-63, HOS-MNNG and K-HOS and mouse cell line MOS-J with increasing doses of zardaverine, Figure S2: cAMP concentration versus DMSO, in Saos-2 and MNNG/HOS after treatment with zardaverine or IBMX and cAMP-specific PDE genes expression, Figure S3: Saos-2 migration speed and wound closure percentage. Table S1: Nucleotide sequences of primers used for qRT-PCR and efficiency for each primer couple.
