# Supplementary material for: Pyridazinone Derivatives Limit Osteosarcoma-Cells Growth In Vitro and In Vivo
Source: Cancers (Basel). 2021 Nov 28;13(23):5992. doi: 10.3390/cancers13235992 (PMC8656549; doi:10.3390/cancers13235992)
Supplement: Supplementary file 1 [file cancers-13-05992-s001.zip › cancers-1443973-supplementary.pdf]

cancers

MDPI

Supplementary Materials:

RNA Purification and Reverse-Transcription

Total RNAs were extracted and cleaned up from Saos-2 and MNNG/HOS with MasterPureTM RNA Purification Kit in accordance with the manufacturer protocol. RNA purity was assessed by measuring the absorbance ratio at 260/280 nm (Nanodrop 2000C, Thermo Scientific, France), which was comprised between 1.8 and 2. Total RNAs (500 ng) were reverse transcribed into cDNA using a high-capacity cDNA reverse transcription kit following the manufacturer instructions.

Transcription products were amplified by qRT-PCR using different primers (Table S1) on a StepOne PlusTM system (Applied Biosystems, Villebon-sur-Yvette, France). After a first denaturation step at 95°C for 10 min, qRT-PCR reactions were performed according to a thermal profile that corresponds to 40 cycles of denaturation at 95°C for 15 s, annealing and extension at 60°C for 1 min. Data collection was performed at the end of each annealing/extension step. The third step that consists in a dissociation process is performed to ensure the specificity of the amplicons by measuring their melting temperature (Tm). Data analysis was performed with the StepOneTM Software v2.3 (Applied Biosystems, Villebon-sur-Yvette, France).

Table S1. Nucleotide sequences of primers used for qRT-PCR and efficiency for each primer couple.

Target gene	Sequences		Efficiency
	Forward primer (5′→3′)	Reverse primer (5′→3′)	
PDE4A	CAGCCCCATGTGTGACAA	ATGGGTGCACAATGTAGTCAATA	1.96
PDE4B	CCTTTCTACACCAGCAGACG	GGATGGCAGCTGCAAAAA	1.95
PDE4D	TTGTCCAGTCTACTCATGTGCTATT	TTGCTGCAAGAATCTCCAAA	1.96
PDE7A	TGTCGGACGTGGGAATTAAG	GTGGACCACCCAAATGA	2.03
PDE7B	GTTTGAGAACCCCGATCAGA	CCGTCTGACCCCTTAGTCG	1.99
PDE8A	GCTTCATGCCACTGCCTATT	GTGCAGCGACCTCATCAAT	1.95
PDE8B	ACAGCATCAACAAGCCAATG	GCAGGGTTGCATTCACAGT	2.09

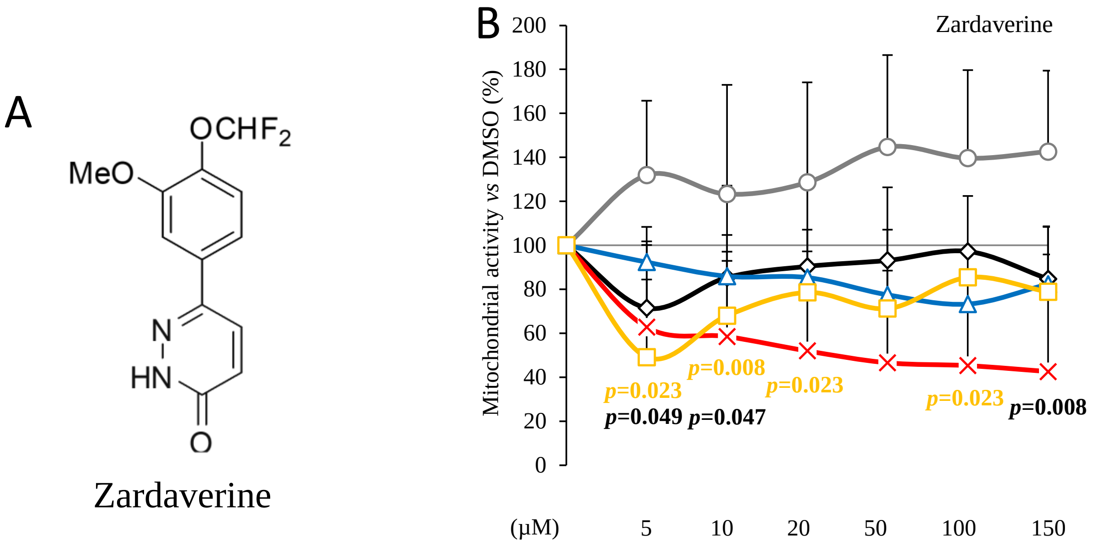

Figure S1. Chemical structure of zardaverine (A). Mitochondrial activity *versus* DMSO on human osteosarcoma cell line Saos-2 (black diamond), MG-63 (yellow square) , HOS-MNNG (red cross) and K-HOS (grey circle) and mouse cell line MOS-J (blue triangle) after 96h of treatment with increasing doses of zardaverine (B). n=7. *p*-values are given relative to DMSO.

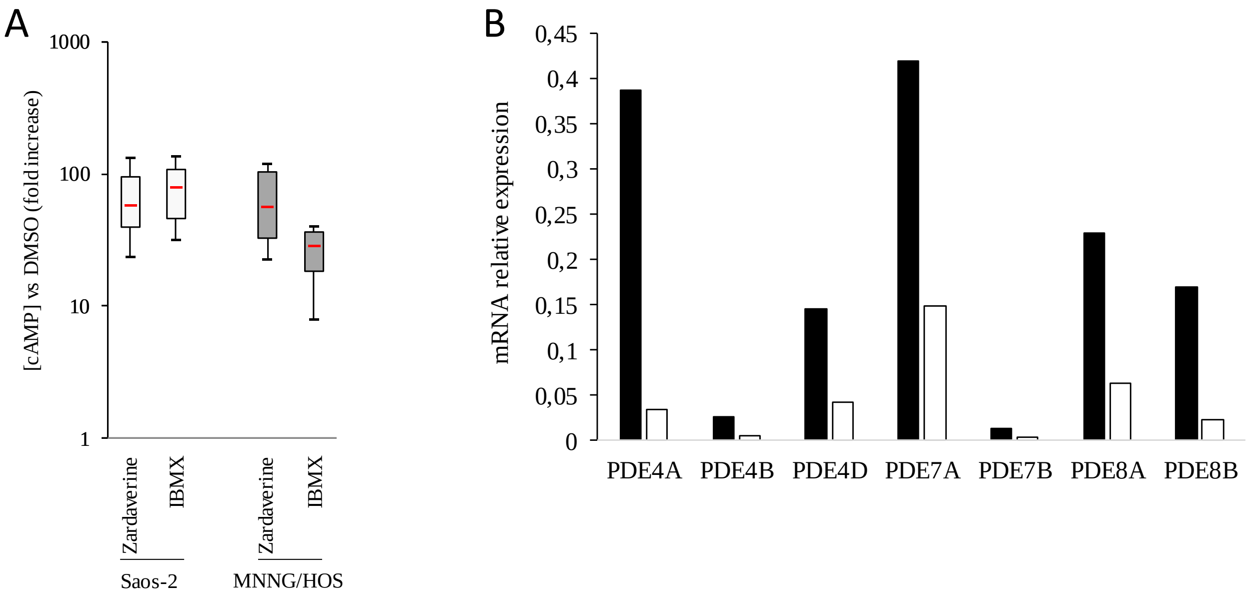

Figure S2. cAMP concentration *versus* DMSO, in Saos-2 and MNNG/HOS after treatment with zardaverine or IBMX (A). cAMP-specific PDE genes expression related to housekeeping gene HPRT-1 in Saos-2 (in black) and MMNG/HOS (in white) (B).

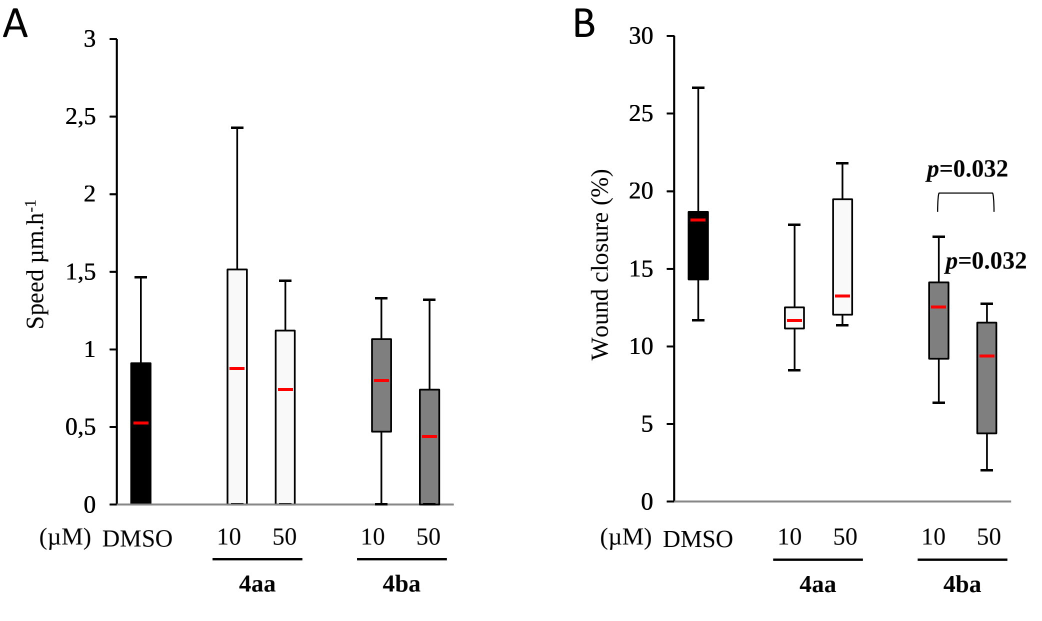

Figure S3. Saos-2 migration speed (A) and wound closure percentage (B). n=7. *p*-values are given relative to DMSO on box-plots, the brackets indicate the conditions compared.
